# Editorial: NCDs – Core Challenge of Modern Day Health Care Establishments

**DOI:** 10.3389/fpubh.2021.692926

**Published:** 2021-06-18

**Authors:** Mihajlo Jakovljevic, Tarang Sharma, Narimasa Kumagai, Seiritsu Ogura

**Affiliations:** ^1^Institute of Comparative Economic Studies, Hosei University, Tokyo, Japan; ^2^Department of Global Health Economics and Policy, University of Kragujevac, Kragujevac, Serbia; ^3^Evidence to Policy, Copenhagen, Denmark; ^4^Seinan Gakuin University, Fukuoka, Japan; ^5^Hosei University, Tokyo, Japan

**Keywords:** non-communicable diseases, costs, health economics, health expenditure, spending, health financing, emerging markets

We have seen the global burden of disease shift significantly from communicable diseases to non-communicable diseases (NCDs) over the past few decades (1). This is happening partly due to the changing demographics with the aging of the population. The third demographic transition or the “Silver Tsunami” has historically began in the Western nations. Later on it has spread worldwide to become a global phenomenon. Another driver of NCDs incidence rise is changing lifestyles of people making cardiovascular diseases the biggest cause of mortality worldwide. There has also been a steady rise in deaths due to cancers, road traffic accidents, and mental health conditions (2). All of these disorders and injuries now feature among the top 10 causes of death globally. This has also meant a so-called “double burden” for low and middle-income countries (LMICs) where both communicable diseases and NCDs coexist together (3).

It was predicted that by 2020 seven out of 10 deaths in developing countries will be attributed to NCDs. Thus, now the significant reallocation of funds is needed to effectively support the treatment and management of these populations in the future. There is an ongoing shift to a chronic disease management model from acute care (4). Such a profound evolution of medical care demand, has serious economic implications and needs a complete re-shaping and re-configuring of healthcare financing mechanisms within most contemporary health systems in the upcoming 2020s (5). Significant challenges are foreseen in LMICs where infrastructure for care is still primarily driven by the infectious disease model and as long-term continued care is needed for the treatment of NCDs, is still at odds with the out-of-pocket payment models common in LMICs, which presents serious challenges (6). It is expected that this transition in health care demand may push developing countries further into poverty (7). Possible exit reform strategies assume development of brand new public funding mechanisms and appropriate public-private partnerships (8). To make the challenge of effective long-term reform even harsher, the ongoing pandemic itself has put these national systems to the test of fiscal sustainability.

This coupling of NCDs with communicable diseases has become even more evident with the COVID-19 pandemic where increased anxiety and mental health problems related to income and job loss were seen in Thailand (9), increased suicidal attempts in Austria (10) and increased anxiety and depression globally (11). There has also been an increase in social economic status disadvantage where the poor will disproportionately bear the long-term economic effects of this pandemic (12) and so health systems need to ensure equity in access to care and services.

The manuscript submission entitled: “Avoidable Cancer Mortality in Germany Since Reunification: Regional Variation and Sex Differences” dealt with the “traditional” East-West gap in preventable cancer mortality. It appears that it is still evident in males, while more regional diversity is obviously present in avoidable cancer mortality in women. North-south divide in avoidable cancer mortality shapes the future trends in Germany Westerman and Mühlichen.

Considering the key factors underlying NCDs such as income inequality and insecure employment, much attention must be paid to unhealthy commercial industries (UCIs) producing tobacco, alcohol, and low-nutrient foods. NCDs should be reframed as the product of a complex system which includes UCIs (13).

Contribution by Arora et al. “The Indian Bidi Industry: Trends in Employment and Wage Differentials,” explored trends in employment and wages in the bidi industry using secondary data from the National Sample Survey Office (NSSO) and the Central Statistics Office (CSO), Government of India–the Annual Survey of Industries (ASI), and Enterprises Survey. Conclusive remarks claim that Bidi workers earn much less compared to workers in other manufacturing industries and are subject to income inequality (Arora et al.).

Ksatri et al. conducted extensive research: “Prevalence and Patterns of Multimorbidity Among Rural Elderly: Findings of the AHSETS Study.” Their observation was that in India, the proportion of older population is projected to increase from 8% in 2015 to 19% in 2050 and a third of the country's population will be older adults by end of the century. Their findings reveal overall prevalence of multimorbidity of 48.8% of which dyads (25%) were the most common form, followed by triads (15.2%) (Kshatri et al.).

The next contribution coming from India has been dealing with corona pandemic. Pati et al. claim that continuation of preventive and management services of NCDs in tandem with COVID-19 containment measures should additionally include self-care and multimorbidity literacy toward patient activation. This research from the Khurda district of Odisha, revealed a higher presence of multimorbidity in younger population (40–60 years). Authors concluded that region-specific pandemic healthcare preparedness plans should necessarily incorporate measures to reduce risk of infection with mental healthcare and NCD management elements, while resuming economic-activity (Pati et al.).

Turrini et al. have published another surprisingly interesting contribution within the Topic in March 2021. Authors propose synergistic collaboration between CREA Research Center for Food and Nutrition and the Italian National Health Institute (ISS) to be promoted and supported by the Italian Ministry of Health. They believe such a health policy approach would support setting up a nutritional surveillance system (Turrini et al.).

Pati et al. have studied 500 adult patients admitted to the psychiatric clinic throughout cross-sectional study design over a period of 6 months in a college hospital in Odisha, India. They used a validated structured questionnaire, “multimorbidity assessment questionnaire for psychiatric care” (MAQ-PsyC) for data collection. Among psychiatric morbidities, mood disorders incurred the highest out of pocket expenditure (OOPE was $93.43). It appeared that psychiatric illnesses had a significant interaction effect on the association between multimorbidity and high medical expenditure. This study has revealed multimorbidity as being highly prevalent in psychiatric patients associated with significantly high healthcare utilization and medical expenditure.

Simic et al. have considered the cost of illness and budget impact estimates related to preventive examinations for ischemic heart disease in active-duty military personnel in Serbia. Periodic health-examination screening program in military personnel enabled not only discovery of patient with newfound arterial hypertension but also regular monitoring of those who were already on antihypertensive therapy, significant savings of €690.58 per patient and €2,026.67 per patient could be achieved, respectively (Simic et al.).

In Japan, a new national consensus is emerging on the need to strengthen our healthcare system against pandemics. Present healthcare system, almost completely specialized in NCD care, was instantly incapacitated when Covid-19 struck Japan. Authorities did not have enough trained physicians, nurses, technicians, medical supplies, testing equipment to cope with the sudden surge of Covid-19 patients. These deficiencies resulted in failures in outpatient and inpatient care in urban areas. In retrospect, Japanese policy makers have not paid sufficient attention to recent close calls of pandemics such as SARS, MERS or avian flu. Thus, this is an excellent example of the large high-income OECD nation, hosting a cutting-edge medical technology, but still facing a challenge beyond NCDs (14).

Throughout this Topic life cycle it became appearant that global pandemic of non-communicable diseases continues as a new tide. This time most of the global burden of these chronic, mostly incurable and expensive-to-treat diseases shall move toward the Global South (15). We anticipate that most of associated workload for the national health sectors in terms of consumer demand for medical services and goods and associated spending will gradually move toward LMICs regions and Emerging markets in particular. How the challenge of fiscal sustainability will be met, remains to be seen during the upcoming 2020s.

## Author Contributions

MJ has prepared the manuscript draft while TS, NK, and SO have revised it for important intellectual content. All authors contributed to the article and approved the submitted version.

## Conflict of Interest

The authors declare that the research was conducted in the absence of any commercial or financial relationships that could be construed as a potential conflict of interest.
